# Correction: Evans et al. Survival-Larval Density Relationships in the Field and Their Implications for Control of Container-Dwelling *Aedes* Mosquitoes. *Insects* 2023, *14*, 17

**DOI:** 10.3390/insects16121210

**Published:** 2025-11-28

**Authors:** Katherine G. Evans, Zoey R. Neale, Brendan Holly, Cecilia C. Canizela, Steven A. Juliano

**Affiliations:** School of Biological Sciences, Illinois State University, Normal, IL 61790-4120, USA

## Error in Table

In the original publication [1], there was a mistake in **Table 3** as published. **The 6th line of the body of the table reported parameter estimates from an earlier, incomplete count of survivors.** The corrected **Table 3** appears below. The authors state that the scientific conclusions are unaffected, as estimate *d* is not significantly different from 1.0, leading to the prediction of compensatory mortality. This correction was approved by the Academic Editor. The original publication has also been updated.

## Figures and Tables

**Table 3:** Density manipulation experiment results. The estimated parameter values for the non-linear regression (Equation (1)) are reported for each experiment, respectively. The interpretations of the results are based on the parameter *d* and are reported in the last column. *d* < 1 additive mortality; *d* = 1 compensation; *d* > 1 overcompensation. N/A indicates that confidence intervals for the associated parameter could not be estimated, usually because the solution converged to an estimate of the upper bound for the parameter (i.e., FLSuburb18-2, FLCemetery19-1, and FLCemetery19-1 experiments).

Experiment Name	Target Species	*a*	95% CI	*K*	95% CI	*d*	95% CI	Interpretation
ILForest17	*Ae. triseriatus*	0.663	[0.596, 0.731]	187.4	[169.0, 205.9]	5.9096	[4.6543, 7.1649]	Overcompensation
ILForest18	*Ae. triseriatus*	0.445	[−0.816, 1.707]	500	[−15,885, 16,885]	−0.1693	[−0.6292, 0.2906]	Additive mortality
MOForest18	*Ae. albopictus*	0.605	[0.5669, 0.6426]	476.8	[457.0, 496.7]	5.4821	[3.8778, 7.0865]	Overcompensation
ILCemetery19	*Ae. albopictus*	0.944	[0.104, 1.283]	137.2	[−272.0, 546.5]	0.6269	[−0.0295, 1.2833]	Additive mortality
FLSuburb18-1	*Ae. aegypti*	0.826	[0.466, 1.186]	250.9	[48.4, 451.6]	1.1658	[0.4167, 1.9150]	Compensation
FLSuburb18-2	*Ae. aegypti*	0.635	[0.379, 0.890]	167.4	[95.7, 239.8]	1.8690	[0.0219, 3.7161]	Compensation
FLCemetery19-1	*Ae. aegypti*	1	N/A	400.2	[−222.5, 1023.0]	0.3976	[0.03373, 0.7614]	Additive mortality
FLCemetery19-2	*Ae. aegypti*	1	N/A	153.8	[122.8, 184.9]	1.0134	[0.5263, 1.5005]	Compensation

## References

[1] Evans K.G., Neale Z.R., Holly B., Canizela C.C., Juliano S.A. (2023). Survival-Larval Density Relationships in the Field and Their Implications for Control of Container-Dwelling Aedes Mosquitoes. Insects.

